# An insight into synthesis and antitumor activity of citrate and gallate stabilizing gold nanospheres

**DOI:** 10.1038/s41598-023-29821-4

**Published:** 2023-02-16

**Authors:** Mohamed M. Fathy, Abdo A. Elfiky, Yousef S. Bashandy, Mayar M. Hamdy, Ahmed M. Elgharib, Ibrahim M. Ibrahim, Rana T. Kamal, Ahmed S. Mohamed, Anan M. Rashad, Ola S. Ahmed, Yomna Elkaramany, Youssef S. Abdelaziz, Fatma G. Amin, Jehane I. Eid

**Affiliations:** 1 Biophysics Department, Faculty of Science, Cairo University, Giza, Egypt; 2 Biotechnology and Biomolecular Chemistry Department, Cairo University, Giza, Egypt; 3 Virology and Immunology Unit, Cancer Biology Department, National Cancer Institute, Cairo University, Giza, Egypt; 4 Botany and Microbiology Department, Faculty of Science, Cairo University, Giza, Egypt; 5 Physics Department, Faculty of Science, Alexandria University, Alexandria, Egypt; 6 Zoology Department, Faculty of Science, Cairo University, Giza, Egypt

**Keywords:** Biophysics, Molecular biology

## Abstract

Both gallic and citrate are well-established antioxidants that show promise as new selective anti-cancer drugs. Gold nanoparticles (AuNPs) as well can be developed as flexible and nontoxic nano-carriers for anti-cancer drugs. This article evaluating the efficiency and biocompatibility of gallic acid and citrate capping gold nanoparticles to be used as anti-cancer drug. The biosafety and therapeutic efficiency of prepared nano-formulations were tested on Hela and normal BHK cell line. Gold nanospheres coated with citrate and gallate were synthesized via wet chemical reduction method. The prepared nano-formulations, citrate and gallate coated gold nanospheres (Cit-AuNPs and Ga-AuNPs), were characterized with respect to their morphology, FTIR spectra, and physical properties. In addition, to assess their cytotoxicity, cell cycle arrest and flow cytometry to measure biological response were performed. Cit-Au NPs and Ga-Au NPs were shown to significantly reduce the viability of Hela cancer cells. Both G0/G cell cycle arrest and comet assay results showed that genotoxic effect was induced in Hela cells by Cit-Au NPs and Ga-Au NPs. The results of this study showed that Cit-Au NPs and Ga-AuNPs inhibit the growth of metastatic cervical cancer cells, which could have therapeutic implications.

## Introduction

Normal cells are known to have defined life span then the cell start to undergo controlled death condition which is apoptosis. The name "apoptosis" (a-po-toe-sis) was first used in a now-classic study by Kerr, Wyllie, and Currie in 1972 to characterize a physically different form of cell death^1^. Apoptosis is a natural process that happens throughout growth and aging in order to keep cell populations in tissues stable. And also, as a protective process, such as in immunological responses or when diseases or toxic chemicals destroy cells^2^. Wide range of normal and pathological stimuli can cause apoptosis. Such as DNA damage from radiation or chemotherapy medications, which can trigger apoptotic cell death via a p53-dependent mechanism. While in other cases some hormones, such as corticosteroids, may cause apoptotic death in some cells (such as thymocytes). Finally, apoptosis is a coordinated, energy-dependent process that entails the activation of a family of cysteine proteases known as "caspases" and it is stimulated by multiple factors^3^.

In the year 2000, Hanahan and Weinberg published their influential review: the hallmarks of cancer^4^.divided into six main hallmarks (referred to as Hallmarks I), including self-sufficiency in growth signals, insensitivity to anti-growth signals, evading apoptosis, limitless replicative potential, sustained angiogenesis, and tissue invasion and metastasis. An updated evaluation a decade later^5^. And since then, studying the ability of different compounds on cancer cells to induce apoptosis gained much interest to be used as a therapeutic approach.

In this paper we tested the cytotoxic and genotoxic induction capacity of gold nanoparticles (AuNPs) in two compounds and evaluated their toxicity levels on both cell lines to understand the best of them on our HeLa cell line. We are working on HeLa cell line, which is cervical cancer cells, the fourth most frequent malignancy in women. High-risk human papillomaviruses (HPV) is the reason behind most cases which is spread by sexual contact, infections can be avoided by primary prevention (HPV vaccines) or second prevention (screening for and treating precancerous lesions) yet, persistent infections lead to cervical cancer.

Treatments for cervical cancer are multiple such as what’s recommended by the National Comprehensive Cancer Network (NCCN), combination chemotherapy regimens with a platinum-containing drug and a taxane, with or without bevacizumab (chemotherapy doublet + bevacizumab)^6^, or Pembrolizumab, which is authorized for patients whose tumors express programmed death-ligand 1. However, with continuous initiatives to discover novel medicines for patients who progressed in this environment, the landscape of r/mCC (recurrent-metastatic cervical cancer) treatment is fast altering in order to find the best drugs to inhibit cancer pathways and return to normal conditions^7,8^. Given the global growth in cancer incidence and the adverse effects of present treatment methods (such as discomfort, sleep issues, hair loss, and bleeding), it appears imperative to develop new therapeutic procedures and replace old ones.

Nano-medicine was a new approach for a higher degree of efficacy in treating cancers with least possible side effects^9^. Gold nanoparticles (AuNPs) are considered one of the most dominant systems for many biological applications such as drug delivery and cancer treatment because of their ease in preparation, anti-inflammatory, antioxidant properties, biocompatibility and many possibilities of bio-conjugation^6^. They can have a wide range of sizes and can be relatively small to facilitate its interaction with tissues and cells in many biological systems^7^. Their surface can be easily modified and functionalized with different molecules, allowing for diversity of new drug delivery systems and cancer treating methods^8^. The cytotoxicity of gold nanoparticles depends on the surface modification used and the type of cell that is being targeted, as they can be more resistive or more sensitive to AuNPs treatment^10^. And to enhance the efficacy of the gold nanoparticles drugs they were incorporated with other molecules to enhance their results and those molecules vary as well depending on the targeted cells and desired results and mechanism.

Gallic acid (3,4,5-trihydroxybenzoic acid) is a strong polyphenolic molecule that has been observed to have anti-carcinogenic effects both in vitro and in vivo. Most importantly, gallic acid has a cytotoxic property that is very selective against a variety of tumor cells including lung, colon and prostate cancer cells^11^. Mechanisms that are used by gallic acid (GA) to inhibit carcinogenesis and tumor development have been observed by many studies including inhibition of metastasis^12^; inhibition of cell proliferation, tube formation, migration and invasion^13,14^; preventing of angiogenesis^15^; induction of apoptosis. Citric Acid was also used as a capping agent with gold nanoparticles on Hela cells at different concentrations. It was proved that 20 nm of Cit-Au NPs were of very low cytotoxicity that it was administrated directly to the blood stream of cervical cancer patients that it was used as an alternative drug^16^.

In this paper we compared the cytotoxic and genotoxic induction done by Cit-Au NPs and Ga-Au NPs in different concentrations on both normal and cervical cancer cells to evaluate either they can be used as alternative drugs for the recent treatments or not.

## Materials and methods

### Materials

Hydrogen tetrachloroaurate (HAuCl_4_.3H_2_O), Trisodium citrate dihydrate (HOC) (COONa) (CH_2_COO-Na)_2_(2H_2_O), sodium hydroxide (NaOH), and Gallic acid (GA) were obtained from Sigma-Aldrich (St. Louis, MO, USA). All other chemicals were of analytical grade and used without other purification.

### Preparation of gold nanoparticles

Citrate-capped Gold nanoparticles were prepared by citrate reduction method^17^. A flask containing a 100 ml solution of 1 mM HAuCl_4_ in was boiling in a closed system while being stirred for 30 min. Five ml of 78 mM of trisodium citrate solution was added quickly and allowed to stir for another 15 min. citrate is used as a reducing and capping agent that covering gold atoms forming Cit-AuNPs. The color of the solution is changed gradually from yellow to grey, purple then stabilized at the wine-red color. Due to the negative-charged citrate capping, the nanoparticles repelled from each other preventing the formation of aggregation.

The synthesis of gallic acid capped gold nanoparticles (Ga-Au NPs) was carried by chemical reduction method of hydrogen tetrachloroaurate salt in a liquid phase^18^. A solution of 1 mM of HAuCl_4_ (50 ml) was prepared. Then its pH was adjusted to 11.6 by adding 450 µl of 0.5 M NaOH. Gallic acid solution of 38.8 mM was heated to 40 °C. Then 0.1 ml of gallic acid solution was added to the 10 ml of HAuCl_4_ and stirred in a closed system for 15 min. The color of the solution is changed gradually from yellow to wine-red color.

To identify the amount of gallic acid and trisodium citrate that coated each formulation, the prepared colloids were centrifuged at 15,000 rpm for 20 min. and the supernatant were analyzed with HPLC to determine the free (unloaded) gallic acid and trisodium citrate concentration. Then the encapsulated concentration of gallic acid and trisodium citrate were calculated from the flowing equation.

$$\mathrm{Encapsulated\,concentration \%}=\frac{\mathrm{Total\,added\,concentration}-\mathrm{Free\,concentration}}{\mathrm{Total\,added\,concentration}} \times 100$$

### Characterization of Cit/Ga capped AuNPs

The physiochemical properties of the synthesized AuNPs were characterized using different analyzing tools. The surface plasmon resonance peaks were determined from their absorption spectra that were assessed via UV–Vis spectrophotometer at visible light range of 450–700 nm with distilled water as a blank. Prepared nanoparticles morphology and size distribution were carried by transmission electron microscope (TEM) (Jeol, Tokyo, Japan). A drop of the sample was applied to a copper grid, the excess solution was removed by a filter paper, and allowed to dry in air before examination Particle size distribution of the prepared nanoformulations were performed from TEM images using imagej software^19^. The hydrodynamic diameter and zeta potential measurements of nanoparticles were carried by DLS instrument Zetasizer (Malvern instruments, UK). Dynamic light scattering (DLS) is a technique used for studying the diffusion behavior of molecules in solutions and calculate the hydrodynamic radii from their scattering pattern that depending on the diffusion coefficient of the colloidal nanoparticles^20^. The attachment of citrate and gallic on gold nanoparticles surfaces was studied by FTIR spectroscopy. The sample was deposited in KBr disks and was recorded on a NICOLET 6700 FTIR Thermo scientific spectrometer with a scanning range of 400–4000 cm^−1^, speed of 2 mm/s, a resolution of 4 cm^−1^ at room temperature.

### Cell culture

HeLa and BHK cell lines were obtained from National Oncology Institute, Egypt. The cells were cultured using Roswell Park Memorial Institute-1640 (RPMI-1640) medium RPMI medium supplemented with 10% (v/v) FBS, antibiotics (streptomycin 10 l g/ml, penicillin 100 U/ml). Cells were trypsinized, sub-cultured and allowed to grow till confluency. Cells were maintained at 37 °C in 5% CO2 atmosphere in a humidified incubator.

### Cell viability measured by MTT assay

Different concentrations of AuNPs either free or capped with Gallic acid or Citrate were tested on HeLa cell line and BHK cell line to evaluate the toxicity by MTT [3-[4, 5-dimethylthiazol-2-yl]-2,5-diphenyltetrazolium bromide]- based colorimetric assay. Hela cells (1 × 10^4^) cells were seeded wells of a 96-well microtiter plate and incubated for 24 h. Then, the cells were exposed to different concentration of AuNPs (10–500 lg/ml) at (37 °C, 5% CO_2_ for 24 h incubation). After incubation, cells were washed with PBS, a concentration of (0.5 mg/ml) of MTT dye in each well and allowed in the incubate dark at (37 °C and 5% CO_2_ for 4 h). Finally, 100 ml of dimethyl sulfoxide (DMSO) was added to dissolve the purple formazan crystal in the reaction. The optical density (OD) was determined at 570 nm in an ELISA plate reader r (SpectraMax M5- Molecular Devices, USA).

### Cell cycle analysis measured by flow cytometry

Cell cycle analysis was carried out using A.C Martinez-torrez protocol with some modifications^21^. For each well 10^5^ cells were added and allowed to culture for 24 h, then they were treated with different concentrations of AuNPs. The IC50 concentration obtained for each cell type and nano-formulations. Cells were then trypsinized, washed twice with PBS and centrifuged at 500×*g*. The cell pellet was reconstituted in PBS, fixed with cold ethanol (70% v/v), and stored at 4 °C for the duration of the next day. Later on, cells were rinsed in cold PBS and stained for 30 min at 37 °C in a dark water bath using Propidium Iodide (50 g/ml), RNase (40 g/ml), 0.1% sodium citrate, and 0.03% Triton X 100. Then, a BD FACS Verse flow cytometer was used to examine 30,000 events from each sample. By using the gate for doublet elimination during analysis, aggregates were eliminated. Using the software program (Verity Software House Inc., USA)^22^, the proportion of cells in the G0/G1, S, G2/M of the cell cycle was calculated.

### Comet assay

DNA damage was evaluated using Single cell electrophoresis/Comet assay following N.P. Singh protocol^23^, Alkaline comet assay was picked over neutral assay since its of higher sensitivity to both single and double breaks^24^. 2D cultured cells which was previously treated with AuNPs in three different concentrations were prepared for the assay by trypsinizing and re-suspending them in fresh media to form a solution ready for the assay. Samples were separated and labelled in details. For each sample, we took 5 µl of the suspension and mixed them with 75 µl of Low Melting Point Agarose (250 mg in 50 ml PBS) (Sigma A9414).

Sample smear was formed on the Pre-coated slides with a uniform monolayer of 1% (500 mg in 50 ml PBS) Normal Melting Point Agarose (NMA) (HiMedia RM273). Then, slides were let to dry and stored in cold lysing solution (10 mM Tris, 100 mM EDTA, 2.5 M NaCl, PH = 10) (covered in opaque bottles to protect the cells from excessive DNA damage that could arise from the florescence light) at 4 °C for 3 h.

For electrophoresis, slides were put in the electrophoresis tray, covered with the electrophoresis buffer (10 N NaOH, 200 mM EDTA, PH = 13) and it was adjusted on Volt = 24 V, Current = 300 Milliamperes, time = 30 min. After completion, slides were removed, air dried then neutralized for 15 min in the neutralizing solution (0.4 M Tris, PH = 7.5, stored in Room Temperature), and the step was repeated another two times. Then slides were dehydrated by 10% ethanol by dipping them in ethanol for 15 min then removed, dried and step repeated for 2 times. Finally, the slides were hydrated before staining by dH_2_O and then stained with 1X Ethidium Bromide and visualized to check DNA damage. Each slide was imaged using an epi-fluorescent microscope set to a 200 × magnification, and fifty comet nuclei were examined for each sample using CometScore 2.0 software^25^.

### Measurement of cellular uptake of Au NPs using ICP-OES

Inductively Coupled Plasma Optical Emission Spectroscopy (ICP-OES) is a technique used to quantify the composition of elements in samples by depending on plasma and spectroscopic methods. The idea is to use plasma to excite electrons and detect their emission spectrum, as each element has its own characteristic emission spectrum. The emission intensity on the wavelength can be measured and converted into a concentration^26^. The advantage of the ICP-OES technique is its ability to detect many elements simultaneously, with low detection limits^27^. The effect of different capping modification on the cellular uptake of prepared nanoparticles were assessed using ICP-OES. After treatment with gold nanoparticles, they were washed twice using PBS. The adherent cells were trypsinized, counted, and stored at − 20 °C. Samples were diluted and delivered in 15 ml falcon tubes. They should be acidified in order to keep metals in solution and to destruct solid materials with the use of HNO_3_ (concentration 1–5%). Furthermore, the samples are digested in water bath at 95 °C. The gold concentration in samples is detected by ICP-OES. ICP-OES is used to quantify the concentration of AuNPs uptake through normal and cancerous cells, treated with Cit-Au NPs and Ga-Au NPs. In order to calculate the number of nanoparticles uptake per cell, the total amount of gold in each sample was retrieved from ICP results in respect to every sample volume. Average number of gold atoms per particle was calculated from the size obtained from TEM and the calculations of gold mass; in order to reach to the total number of nanoparticles in each sample. The total number of nanoparticles was divided by the number of cells counted after treatment^28^.

### Data analysis

Data of in vitro assays was analyzed GraphPad Prism 5 software to determined IC50 values^29^. All data obtained were statistically analyzed using the analytical software package (IBM-SPSS)^30^ version 22. According to Kolmogorov–Smirnov test, the data were normally distributed. Duncan’s test was utilized to study the similarity among the different groups. Data were displayed as mean ± standard error of mean.

## Results

### Characterization of Cit/Ga capped AuNPs

**Figure 1:**
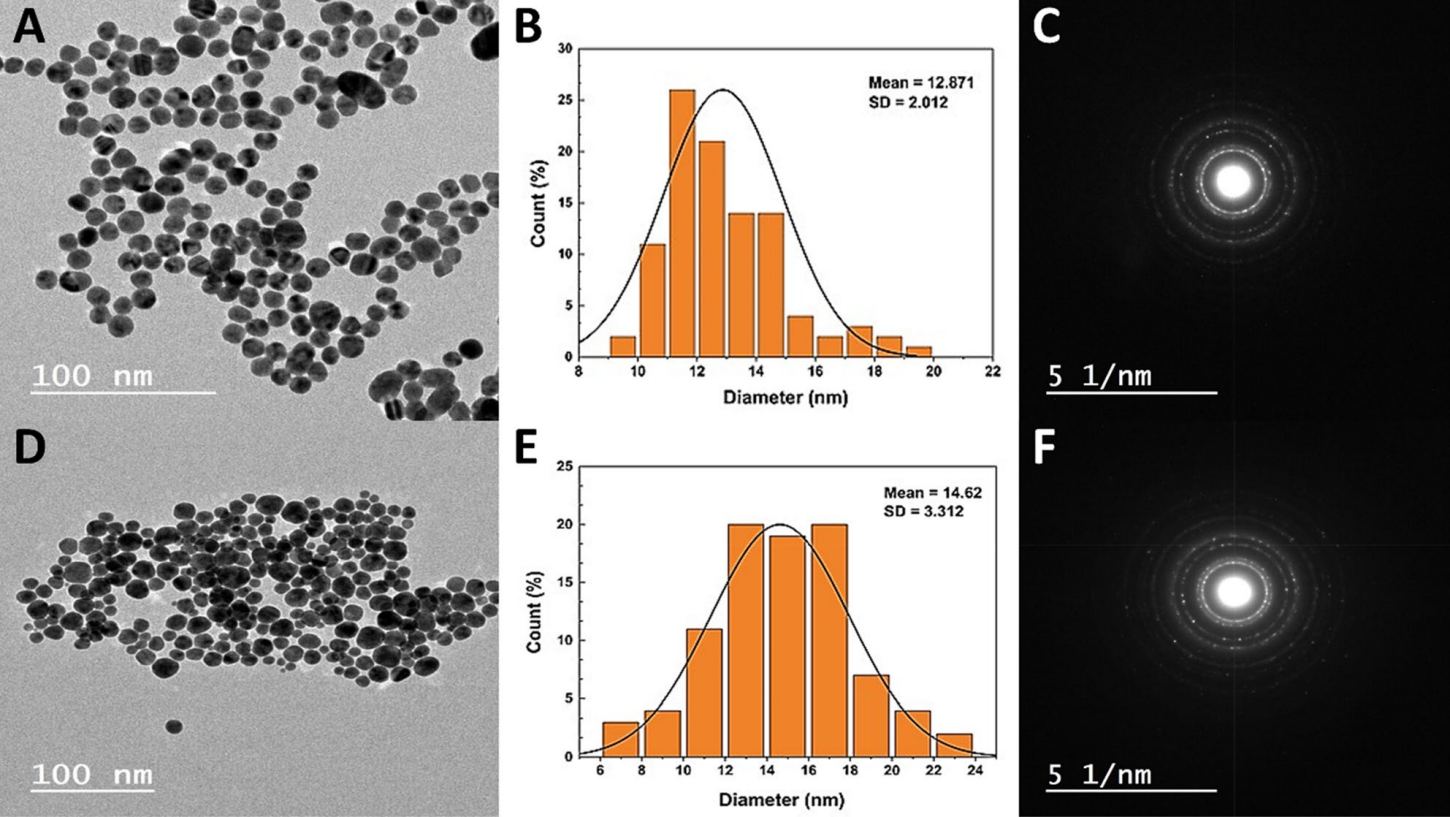
TEM image of Cit-Au NPs (**A**), their particle size distribution (**B**) and their electron diffraction pattern (**C**). TEM image of Ga-Au NPs (**D**), their particle size distribution (**E**) and their electron diffraction pattern (**F**).

**Figure 2:**
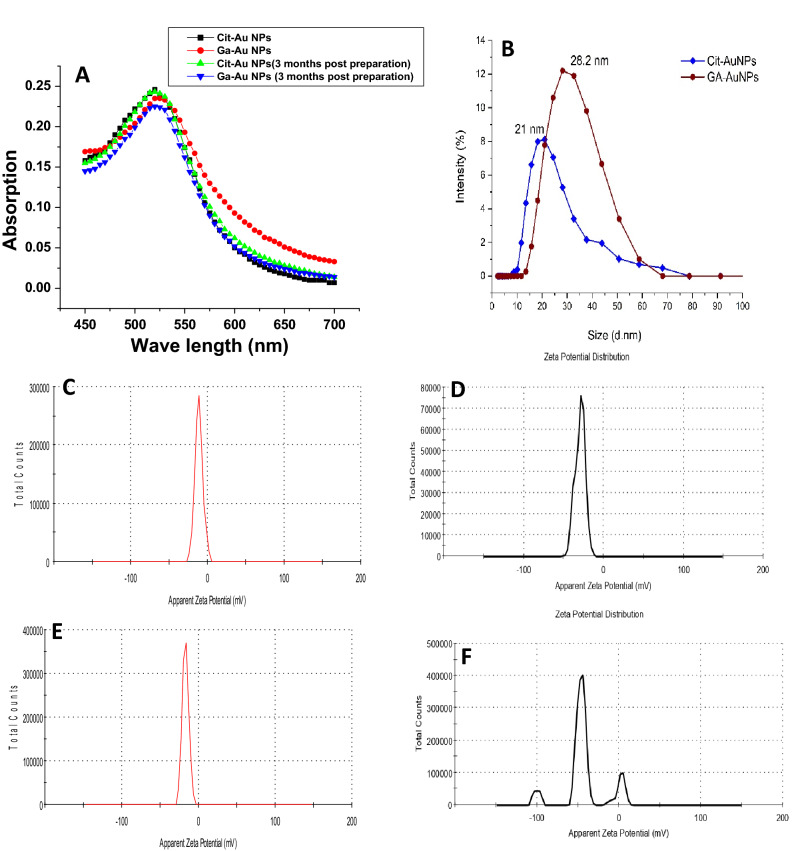
The UV–VIS absorption spectra of freshly and three months post synthesized Cit-Au NPs and Ga-Au NPs (**A**), hydrodynamic size distribution for Cit-AuNPs and Ga-AuNPs (**B**), Zeta potential spectra of Cit-Au NPs and Ga-Au NPs at acidic 5.6pH (**C**,**E** respectively), and Zeta potential spectra of Cit-Au NPs and Ga-Au NPs at normal physiological 7 pH (**D**,**F** respectively).

The morphology and size of synthesized Cit-Au NPs and Ga-Au NPs were investigated using TEM and electron diffraction technique (Fig. 1A,C,D,F). It’s clear that synthesized Cit-Au NPs and Ga-Au NPs has a spherical shape, and have average sizes of about 12–14 nm. The characteristic surface plasmon resonance (SPR) bands are shown at 520 nm and 525 nm for Cit-Au NPs and Ga-Au NPs respectively (Fig. 2A). To ensure the colloidal stability of prepared nano-formulations, the UV–Vis spectra were assessed after 3 months. The spectra show that there is no noticeable change in band position after 3 months storage, which indicates good colloidal stability of the sample. Dynamic light scattering measurements shows that the average hydrodynamic sizes of the Cit-Au NPs and Ga-Au NPs are 21 ± 4.1 nm and 28.2 ± 6 nm respectively (Fig. 2B). And polydispersity indies (PDI) values are 0.224 and 0.320, respectively (Table 1). The average zeta potential measurements (at pH 5.6, similar to the acidic extracellular microenvironment of tumor^31,32^) revealed that the surface potential of the synthesized NPs were negative with an average value of − 16 ± 2.3 and − 11.1 ± 2.6 mv for the prepared Cit-Au NPs and Ga-Au NPs respectively (Fig. 2C,E). While at pH 7.0, equivalent to the storage condition and normal physiological pH, zeta potential values were − 29.4 ± 3.1 and − 46.2 ± 5.3 mV for the prepared Cit-Au NPs and Ga-Au NPs respectively (Fig. 2D,F). The encapsulation efficiency of gallic acid and tri-sodium citrate loaded to Ga-Au NPs and Cit-Au NPs were found to be 98.5% and 75.8% respectively.

**Table 1:** A Summary of the measured Hydrodynamic diameter and zetapotential of the prepared gold nanoparticles.

Sample name	Hydrodynamic diameter	polydispersity indies (PDI)	Zeta potential value at pH 5.6	Zeta potential value at pH 7
Cit-Au NPs	21 ± 4.1 nm	0.224	− 11.1 ± 2.6 mV	− 29.4 ± 3.1 mV
Ga-Au NPs	28.2 ± 6 nm	0.320	− 16 ± 2.3 mV	− 46.2 ± 5.3 mV

### FTIR results of Cit/Ga capped AuNPs

**Figure 3:**
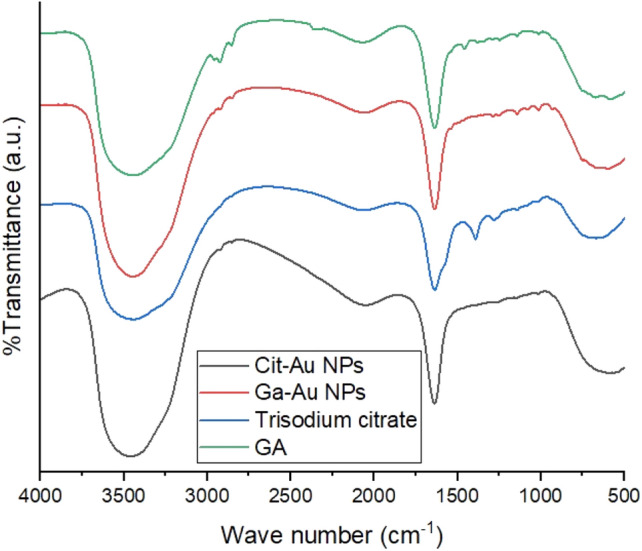
Representation of the FTIR spectrum for gallic acid, trisodium citrate, gallic acid conjugated gold nanoparticles, citric acid conjugated gold nanoparticles.

Figure 3 represents the FTIR spectrum for gallic acid, Citric acid, gallic acid conjugated gold nanoparticles, citric acid conjugated gold nanoparticles. The characteristic C=O stretching at 1637.27 cm^−1^ in gallic acid was detected in gallic acid conjugated gold nanoparticles. Phenolic OH vibration of gallic acid at 3440.38 cm^−1^ was found also in gallic acid conjugated gold nanoparticles which confirms the successful conjugation of gallic acid to the surface of gold nanoparticles. Sodium citrate FTIR spectrum showed the characteristic peaks at 1637 and 1391.39 cm^−1^ of symmetric and anti-symmetric stretching of COO. Citrate capped gold nanoparticles showed only one peak at 1638 cm^-1^ which corresponds to C=O stretching at the citrate capping agent^33^.

### MTT assay

**Figure 4:**
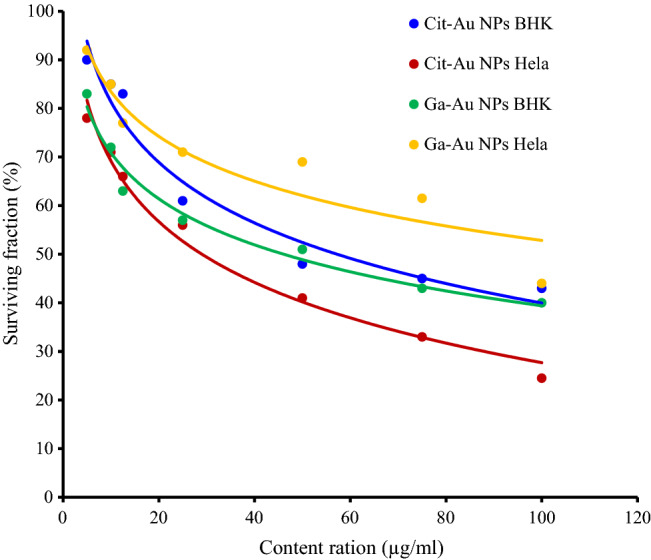
In vitro cytotoxic activity of Cit/Ga capped AuNPs on Hela and BHK cells for 24 h.

After evaluating the NPs characteristics their toxicity on the proliferative ability of Hela cells versus BHK cells was evaluated. Hela cells and BHK cells were treated with the same concentrations for 24 h, and viability was measured by MTT assay. As shown in (Fig. 4), The half maximal inhibitory concentration (IC50) calculated for GA-Au NPs on Hela cells was 91 µg/ml, while that for Cit-Au NPs as shown was 34 µg/ml, the results indicated that Cit-Au NPs exhibited higher cytotoxicity against Hela cells as compared to GA-Au NPs. In fact, Cit-Au NPs and GA-Au NPs produced remarkable Hela cell growth inhibitory effects in a concentration-dependent manner. While cytotoxicity on normal cell line show that GA-Au NPs exhibited lower cytotoxicity to normal BHK cells (IC50 = 52 µg/ml) compared to Cit-Au NPs at which the (IC50 = 45 µg/ml). Cytotoxicity assessment of the capping agents (gallic and citrate) were revealed a nonsignificant toxic effect against both types of cells (Hela and BHK cells) (supplementary material, Fig. S1).

### Flow cytometric cell cycle assay

**Figure 5:**
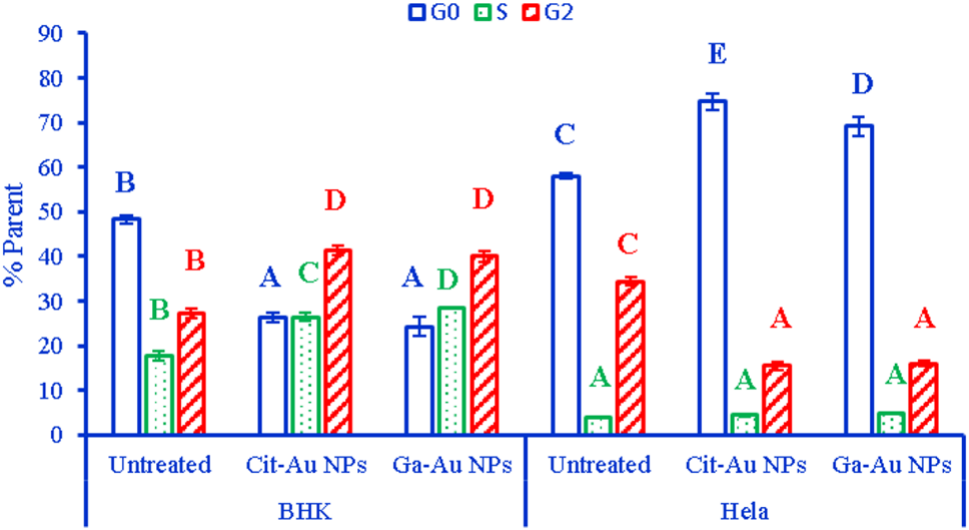
Represents percentage of total of cell cycle stages of BHK and Hela cell lines after treatment with Cit-Au NPs and Ga-Au NPs. Data are presented as mean ± standard error. In the same cell cycle stage, bars marked with different letters are significantly different (P < 0.05), whereas those marked with similar ones are insignificantly different (P > 0.05).

The flow cytometric cell cycle assay was tested with the same two model chemicals (GA-Au NPs and Cit-Au NPs) as in cytotoxicity assay. Proliferative potency of gold nanoparticles in this assay is represented by a change in percentage of cells in S-phase of the cell cycle. For optimization of the flow cytometric assay, the effect of exposure period on chemical-reduced cell proliferation response was examined with concentrations of IC_50_ of the two model chemicals, obtained in the cytotoxicity assay. Analysis of the proliferative effects of Ga-Au NPs and Cit-Au NPs in time clearly showed that greatest responsiveness was obtained after 24 h exposure. Clear G0/G1 arrest was achieved after incubation of Hela cells for 24 h with Ga-Au NPs and Cit-Au NPs, are represented as (69.23% cells in G0/G1, 4.89% cells in S) and (74.71% cells in G0/G1, 4.39% cells in S) respectively as shown in (Fig. 5).

### Comet assay

Comet assay was used to evaluate the DNA damage done on both cell lines at different concentrations of Ga-Au NPs and Cit-Au NPs and it proved that Ga-Au NPs caused more damage to the DNA content of both normal and Hela cells and it was significantly more than that caused by Cit-Au NPs. and this was shown by measuring the tail momentum as shown in Fig. 7, where the tail Momentum (TM) of Hela cells with Ga-Au NPs was larger than it on normal cells and untreated cells. While Cit-Au NPs showed more effect on cancer cells than normal cells, yet it was still less than that caused by Ga-Au NPs.

### Cellular uptake quantification of Au NPs

The uptake of Cit-Au NPs (1.46 × 10^13^ NPs/cell) and Ga-Au NPs (1.17 × 10^13^ NPs/cell) for Hela cells at the concentrations of IC_50_ is smaller compared to the uptake of BHK cells (Cit-Au NPs is 3.13 × 10^13^ NPs/cell and 1.46 × 10^13^ NPs/cell), at their corresponding IC_50_ concentrations, due to the different membrane composition and the difference in membrane surface charge. The plasma membrane of cancer cells is known for their negative charges on the outer leaflet which opposes the internalization of negatively charged nanoparticles, hence the lower uptake in the case of Hela cancer cells^34,35^. The cellular uptake of both Cit/Ga-AuNPs in the Hela and BHK cells after 24 h incubation period, are shown in Table 2.

**Table 2:** Cellular uptake of gold nanoparticles measured by ICP-OES in (µg/ml), and the calculated number of nanoparticles per cell.

Cell line name	Gold treating concentration	No of NPs/cell
BHK	Control (untreated)	0
Hela	Control (untreated)	0
BHK gold/citrate	45 µg/ml	3.13277 × 10^13^
BHK gold/gallic	52 µg/ml	1.46849 × 10^13^
Hela gold/citrate	34 µg/ml	1.46849 × 10^13^
Hela gold/gallic	91 µg/ml	1.17479 × 10^13^

## Discussion

GNPs are very well known for their anticancer properties and tested on various cell lines and especially used on Hela cell lines to decrease their resistance to radiation because of their positive radiosensetizing properties^9^. Various effects can be gained out of AuNPs in their different structures and different incorporations^36^. In this study we assessed the biosafety and therapeutic efficiency of AuNPs when capping with citrate or gallic acid.

The morphology and size of synthesized Cit-Au NPs and Ga-Au NPs were investigated using TEM and electron diffraction technique (Fig. 1). It’s clear that synthesized Cit-Au NPs and Ga-Au NPs has a spherical shape and have average sizes of about 12–14 nm. The TEM images reveal that Cit-Au NPs have a uniform size distribution higher than that of Ga-Au NPs, which supports the results of DLS. In addition, electron diffraction pattern shows that Cit-Au NPs have a higher crystalline degree than Ga-Au NPs.

The characteristic surface plasmon resonance (SPR) bands are shown at 520 nm and 525 nm for Cit-Au NPs and Ga-Au NPs respectively (Fig. 2A). That indicates the successfully formation of gold nanoparticles. It was proved that the UV–Vis spectrum of gold nanoparticles express an SPR with absorption peak that is highly depending on the size, shape and surface modification of the synthetic nanoparticles^37^. For particles smaller than 10 nm, the SPR band is highly damped due to phase changes caused by increased electron surface collisions. The increases in particles size cause red shifts for the SPR band. For particles larger than 100 nm, the band is highly broadened due to existence of higher order electron oscillations^38^.

The hydrodynamic sizes include the main particle size in addition to its hydration shell in an aqueous environment, which can be affected by hydrodynamic conditions and particle agglomeration. The polydispersity index (PDI) is an indication about the degree of homogeneity of nanoparticles size distribution. The PDI values from 0.1 to 0.25 indicate a narrow size distribution, while a PDI larger than 0.5 is a sign for broadened distribution^39^. The Ga-Au NPs show a hydrodynamic size larger than that of Cit-Au NPs with a broader size distribution. This can be referred to the existence of higher surface charge on citrate than that of gallic acid, which repeals citrate particles from gathering on NPs surface and may help in preventing particle agglomeration.

Zeta potential also plays major role in the cellular uptake of nanosystems^16,40^. Negatively charged nanoparticles showed a prolonged blood circulation time, lower liver uptake and high tumor uptake^41^. In contrary, depending on the fact that cancer cellular membrane charge is negatively charged, the positively charged gold nanoparticles were found to be highly absorbed and internalized into breast cancer cells SK-BR-3 compared to negatively charged gold nanoparticles^40,42^. Also, negatively charged gold nanoparticles showed better uptake rather than the neutral gold nanoparticles and this is due to the presence of certain positively charged regions in the cell surface^42^. Negatively charged nanoparticles exhibits lower albumin absorption which in turn can explain their better blood circulation^43^.

Cell cycle regulators are often mutated in the majority of common malignancies, according to assessments of the molecular characteristics of human tumors, and thus regulating the cell cycle in cancer cells could be an effective way to obstacle the growth of tumors Cell^33^. G1 phase is transition phase between the mitosis and S-phase, in which the cell is metabolically active preparing for replication occurs in S- phase. Therefore, cells have the chance to either go through repair processes or follow the apoptotic pathway when the progression of the cell cycle is arrested in the G1 phase because of defect occurred. Apoptosis is regarded as a preventative measure against the advancement of cancer since it is essential in removing the system of the mutated and hyperproliferating neoplastic cells. Our data showed that the treatment of Hela cells with Ga-AuNPs and Cit-AuNP at concentration of 91 µg/ml and 34 µg/ml respectively, have induced G1 phase arrest of cell cycle progression. This suggests that blocking cell cycle progression may be one of the processes by which Ga-Au NPs and Cit-Au NP work to prevent the proliferation of cancer cells and induce apoptotic pathway.

**Figure 6:**
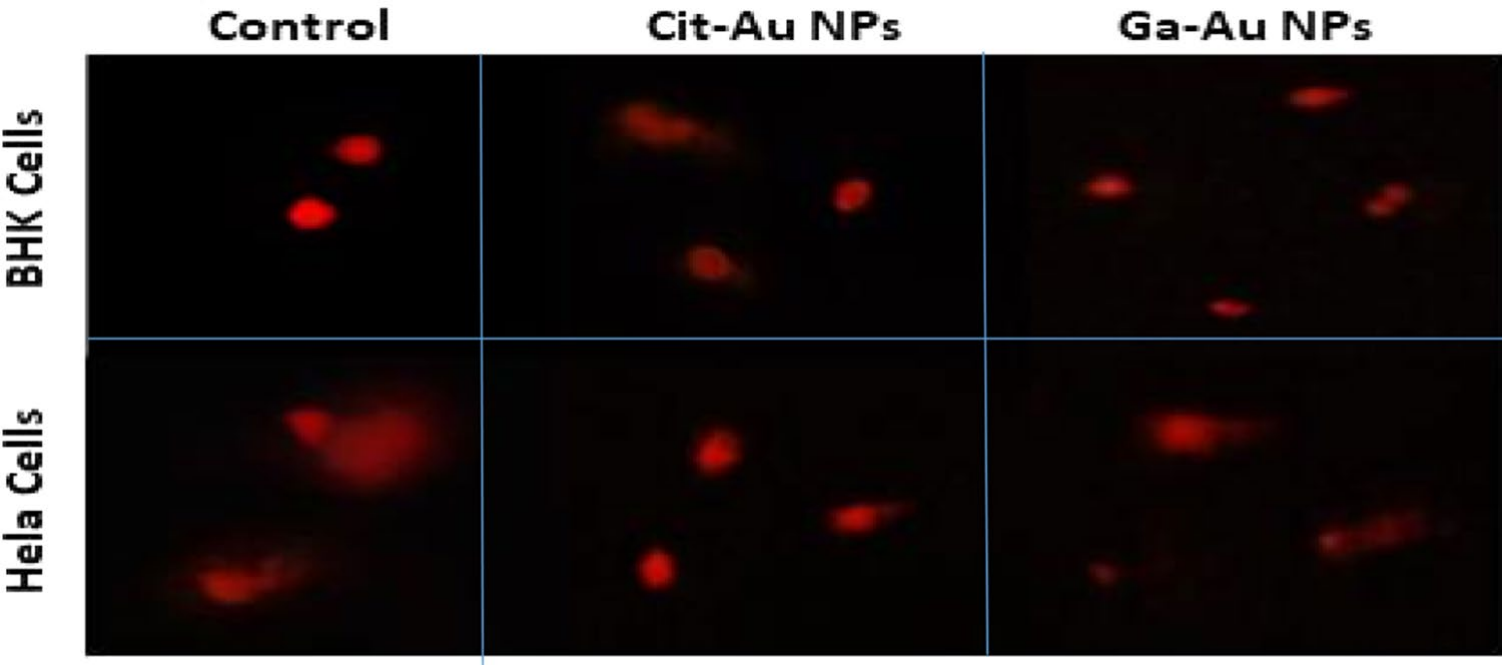
Examples for comet images for BHK and Hela cells before and after treatment with Cit/Ga-Au NPs for 24 h, showing nuclei with intact DNA and damaged DNA.

**Figure 7:**
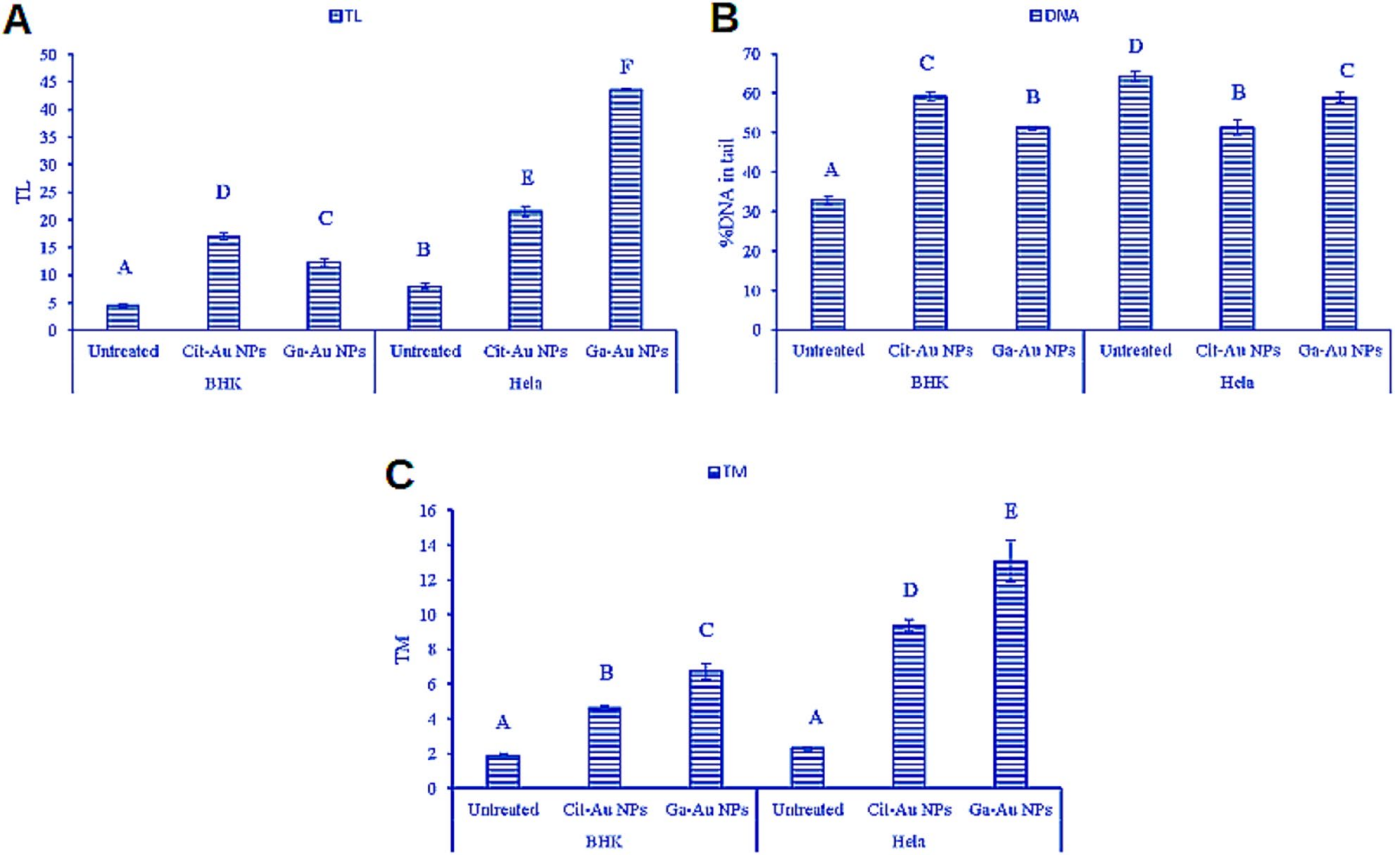
(**A**) Represents tail length (TL), (**B**) percentage of DNA damage in tail (%DNA) and tail moment (TM) of BHK and Hela cell lines after treatment with Cit-Au NPs and Ga-Au NPs. Data are presented as mean ± standard error. Bars marked with different letters are significantly different (P < 0.05), whereas those marked with similar ones are insignificantly different (P > 0.05). In each corresponding treatment, TL of BHK was always significantly lower than that of Hela cells. The TL of Hela cell exposed to Cit-Au NPs was significantly higher than the untreated cells but was remarkably lower than those exposed to Ga-Au NPs.

Endogenous (ROS-induced) or exogenous (UV and other radiation-induced) DNA damage has been demonstrated previously^44^. Both double strand and single strand breaks are the most prominent types of DNA damage^45^. There are several ways that gold nanoparticles (AuNPs) could potentially damage DNA in the nucleus. One possibility is through the generation of reactive oxygen species (ROS), which are chemically reactive molecules that contain oxygen and can be harmful to cells. These ROS can be generated when AuNPs are taken up by cells, and they can cause DNA damage through a variety of mechanisms. Several studies have revealed the capability of AuNPs to induce caspase mediated apoptosis pathway^46–48^.This study measured cell DNA damage using comet assay, which is known for its simplicity and speed^49^, ability to detect DNA damage in a variety of cell types^50^, and qualitative and quantifiable index to evaluate DNA damage^51^. Electrophoresis transports damaged DNA out of the cell, generating a comet tail. Intact cell membrane DNA forms the comet head^52^. The Comet Assay showed DNA damage in Hela cancer cells and noncancer BHK cells after 24 h of Cit/Ga-Au NPs exposure (Figs. 6 and 7). accordingly, Tutty et al. observed that AuNPs capable of damaging HepG2 cell DNA^53^. The aforementioned notion is consistent with the data in this study whereby Cit/Ga-Au NPs resulted in cell cycle arrest and DNA damage that ultimately led to a mechanism of cell death. It is important to elucidate that the specific mechanisms by which GA-Au and Cit-Au NPs may damage DNA are not yet fully understood and will likely depend on a variety of factors, including the size, shape, and surface properties of the NPs, as well as the cell type and the conditions under which the NPs are administered. Further research will be needed to fully understand the mechanisms by which these GA-Au and Cit-Au NPs may damage DNA and to identify any potential pathways that may be involved.

## Conclusion

The findings of this study indicated that Cit-Au NPs and Ga-Au NPs suppress the growth of HeLa cancer cells. Since this indicates that these nanoparticles may have the potential to be used in the field of therapeutics, further research needs to be conducted in this area.

## Supplementary Information

Supplementary Figure 1.

## Data Availability

The datasets used and/or analyzed during the current study available from the corresponding author on reasonable request.
