# Supplementary material for: An insight into synthesis and antitumor activity of citrate and gallate stabilizing gold nanospheres
Source: Sci Rep. 2023 Feb 16;13:2749. doi: 10.1038/s41598-023-29821-4 (PMC9935520; doi:10.1038/s41598-023-29821-4)
Supplement: Supplementary file 1 — Supplementary Figure 1. [file 41598_2023_29821_MOESM1_ESM.docx]

(a)

(b)

Figure (S1): The survival rate (%) of BHK and Hela cell lines after treatment with serial concentrations of gallic acid (a) and citrate (b).
